# Retraction: Clinical and Epidemiological Interventions for Monkeypox Management in Children: A Systematic Review

**DOI:** 10.7759/cureus.r190

**Published:** 2025-08-26

**Authors:** Viral Maru, Usama bin Ghaffar, Anurag Rawat, Mohammed Yunus, Akshayraj K Langaliya, Shubhangi Vyas, Dhaval Mehta, Akshaya Ojha

**Affiliations:** 1 Pediatric Dentistry, Government Dental College and Hospital, Mumbai, IND; 2 Basic Sciences, Majmaah University, Al Majmaah, SAU; 3 Interventional Cardiology, Himalayan Institute of Medical Sciences, Dehradun, IND; 4 Pathology, Imam Abdulrahman Bin Faisal University, Dammam, SAU; 5 Conservative Dentistry and Endodontics, AMC Dental College and Hospital, Ahmedabad, IND; 6 Dentistry, AMC Dental College and Hospital, Ahmedabad, IND; 7 Oral Medicine and Radiology, Narsinhbhai Patel Dental College and Hospital, Sankalchand Patel University, Visnagar, IND; 8 Pediatric Dentistry, Private Practice, Jammu, IND

The Editors-in-Chief are retracting this article due to multiple data discrepancies in the search strategy that cannot be explained. The authors did not respond to multiple requests for clarification. As a result, the journal no longer has confidence in the results of this study.

